# Hepatic lipid accumulation induced by mono-(2-ethylhexyl) phthalate and potential ERBB2-associated inflammatory signaling in NAFLD

**DOI:** 10.3389/fphar.2026.1848121

**Published:** 2026-06-25

**Authors:** Caiqi Huang, Mingzhu Yin, Huimin Liu, Wenwu Jia, Lingchuan Niu, Jia Ming

**Affiliations:** 1 Department of Breast and Thyroid Surgery, The Second Affiliated Hospital of Chongqing Medical University, Chongqing, China; 2 Department of Rehabilitation, The Second Affiliated Hospital of Chongqing Medical University, Chongqing, China; 3 School of Traditional Chinese Materia Medica, Tianjin University of Traditional Chinese Medicine, Tianjin, China; 4 Department of Gastrointestinal Surgery, The Second Affiliated Hospital of Chongqing Medical University, Chongqing, China

**Keywords:** hepatic lipid accumulation, inflammatory signaling, MEHP, NAFLD, network toxicology

## Abstract

**Background:**

The primary active metabolite of di (2-ethylhexyl) phthalate (DEHP), mono (2-ethylhexyl) phthalate (MEHP), has received increasing attention due to its association with metabolic liver injury. However, the mechanisms underlying its involvement in nonalcoholic fatty liver disease remain poorly understood.

**Methods:**

This study employed an integrative approach combining network toxicology, machine learning, and molecular docking analyses to systematically investigate the potential molecular mechanisms of MEHP-related NAFLD and to identify candidate targets potentially involved in disease progression. Immune infiltration analysis and *in vitro* experiments were conducted to further validate the functions of the candidate targets.

**Results:**

Multidimensional analyses identified ERBB2 as a candidate gene worthy of further investigation. Immune infiltration analysis suggested that ERBB2 may be associated with alterations in the hepatic immune microenvironment, whereas molecular docking analysis indicated a potential interaction between MEHP and ERBB2. Further *in vitro* experiments showed that MEHP exposure increased ERBB2 protein expression, accompanied by elevated levels of inflammation-related proteins, including TNF-α, IL-6, and IL-1β. Collectively, these findings suggest that ERBB2 may be related to MEHP exposure-associated inflammatory molecular alterations and regulation of the hepatic immune microenvironment.

**Conclusion:**

From the perspective of inflammation and immune regulation, this study proposes that ERBB2 may be involved in MEHP-induced NAFLD, providing a new theoretical basis for toxicological research on environment-related NAFLD.

## 1 Introduction

NAFLD has become the most common chronic liver disease worldwide and poses an increasingly serious public health burden (Younossi et al., 2016a; Friedman et al., 2018). The disease spectrum can progress from simple hepatic steatosis to non-alcoholic steatohepatitis (NASH), liver fibrosis, and even cirrhosis. Currently, the prevalence of NAFLD in the general population worldwide is as high as 38%, and this proportion is even higher among overweight and obese individuals (Younossi et al., 2016b; Wong et al., 2023; Amini-Salehi et al., 2024). Recent studies indicate that, beyond lipid metabolic dysregulation, chronic inflammation and imbalance in the hepatic immune microenvironment play critical roles in the onset and progression of NAFLD (Browning and Horton, 2004; Lee et al., 2023). Evidence suggests that excessive free fatty acids derived from visceral adipose tissue and chronic low-grade inflammation are considered the two most important drivers of liver injury progression in patients with nonalcoholic fatty liver disease (Milić et al., 2014). Multiple pro-inflammatory cytokines, including tumor necrosis factor-α (TNF-α), interleukin-6 (IL-6), and interleukin-1β (IL-1β), are thought to contribute to hepatic inflammatory responses, insulin resistance, and lipid metabolic disturbances, and are closely associated with NAFLD progression (Tsukamoto, 1999; Fujii and Kawada, 2012; Katsarou et al., 2020).

DEHP, a widely used plasticizer, is commonly found in polyvinyl chloride (PVC) products, where it is used to enhance the material’s flexibility and durability (Chen et al., 2020). It is also a recognized endocrine disruptor (Yin et al., 2025) that can enter the human body through various routes, including medical devices, food packaging, and skin contact, potentially causing adverse effects on metabolic health (Pan et al., 2021; Hahladakis et al., 2018). MEHP is the primary active metabolite of DEHP in the body; it has been detected in the urine of approximately 97.1% of the population, indicating extremely widespread exposure (Koch et al., 2005; Benjamin et al., 2017; Zhang et al., 2024). Notably, as the use of plasticizers has increased, the prevalence of NAFLD has shown a concurrent upward trend, suggesting a possible association between the two (Chen et al., 2024). As early as the 1980s, studies found that MEHP exposure could lead to an increase in liver weight as well as morphological and biochemical changes (Seth, 1982). Recent mechanistic studies have further revealed that MEHP may exacerbate NAFLD by promoting lipid accumulation through inhibition of the JAK2/STAT5 pathway and by damaging liver parenchyma via increased oxidative stress (Zhang et al., 2019). Other studies have shown that MEHP can induce lipid accumulation in hepatocytes by upregulating the Notch pathway, with inflammatory responses potentially playing a key role in this process (Zhang et al., 2023). The above studies indicate that MEHP exposure can induce hepatocellular lipid accumulation, disrupt lipid metabolism, and impair liver function, potentially promoting the development of NAFLD. However, the inflammatory regulatory mechanisms underlying MEHP-induced NAFLD remain poorly understood.

ERBB2 (also known as HER2) is a key member of the EGFR/ERBB receptor tyrosine kinase family and has traditionally been recognized for its roles in cell proliferation, differentiation, and tumorigenesis (Yarden and Sliwkowski, 2001). However, emerging evidence indicates that ERBB2’s functions extend beyond tumor-related regulation and may also involve inflammatory responses and immune microenvironment remodeling (Rahman et al., 2019). Studies have shown that in UV-induced skin injury models, ERBB2 activation contributes to the induction of inflammation-related genes such as IL-1β and Ptgs2, suggesting that ERBB2 signaling may participate in the regulation of canonical pro-inflammatory transcriptional programs (Madson et al., 2006). Furthermore, in ERBB2-overexpressing mouse models, lung tissues exhibit pronounced inflammatory changes accompanied by elevated expression of pro-inflammatory cytokines including TNF and IL-6, further supporting a functional link between ERBB2 and inflammatory responses (Zeng et al., 2012). Dendritic cells (DCs), as critical antigen-presenting cells, play essential roles in maintaining hepatic immune homeostasis and regulating the inflammatory microenvironment (Heras-Murillo et al., 2024). Previous research has demonstrated that ERBB2 activation can promote the upregulation of DC maturation markers CD40, CD80, and CD86, thereby enhancing their immune-activating capacity (Gall et al., 2017). Given that chronic inflammation and hepatic immune imbalance are key mechanisms underlying NAFLD development, it is plausible that ERBB2 may contribute to pollutant-induced hepatic inflammatory responses. However, the role of ERBB2 in MEHP-induced NAFLD and its relationship with inflammatory processes remain poorly characterized.

Accordingly, this study integrates bioinformatics analyses, machine learning, molecular docking, immune infiltration analysis, and *in vitro* experiments to explore the potential molecular mechanisms underlying MEHP-induced NAFLD, with a particular focus on the role of ERBB2 in inflammation. We further assessed the expression of inflammation-related proteins, including TNF-α, IL-6, and IL-1β, to evaluate whether ERBB2 may contribute to MEHP-induced remodeling of the hepatic inflammatory microenvironment. The findings are expected to provide new mechanistic insights into pollutant-associated NAFLD.

## 2 Methods

### 2.1 Data acquisition

Four hepatic transcriptomic datasets from NAFLD patients were retrieved from the GEO database. Specifically, GSE89632 (Arendt et al., 2015) and GSE17470 (Baker et al., 2010) were integrated after batch effect correction using the “sva” R package, yielding a combined dataset of 74 samples, including 28 healthy controls and 46 NAFLD cases. This integrated dataset was primarily used to identify NAFLD-associated differentially expressed genes, which were then intersected with MEHP-related genes to determine candidate MEHP-associated NAFLD-related genes. On this basis, machine-learning-based feature selection was further performed, followed by SHAP interpretability analysis to evaluate the importance of the candidate genes and their contribution to model output. Similarly, GSE126848 (Suppli et al., 2019) and GSE24807 (Feng et al., 2015) were integrated following batch effect removal via the “sva” R package, resulting in 62 samples, including 19 healthy controls and 43 NAFLD cases. This dataset was subsequently employed for the construction of binary logistic regression models and nomograms.

### 2.2 Identification of NAFLD-Associated target networks

Differentially expressed genes (DEGs) between individuals with NAFLD and healthy controls were identified using the “limma” package in R (version 4.5.1). Prior to analysis, the batch-corrected expression matrix was used to calculate the average expression for each gene, and only genes with an average expression greater than one were retained to remove low-expressed or noisy genes. Genes meeting the criteria of an adjusted p-value <0.05 and an absolute log2 fold change (|log2FC|) ≥ 0.585 were considered significantly differentially expressed. To further explore MEHP-related targets in the context of NAFLD, a Venn diagram analysis was performed to determine the intersection between MEHP-associated genes and NAFLD-related DEGs. These overlapping genes were subsequently considered candidate targets potentially mediating MEHP-induced NAFLD progression.

### 2.3 Network toxicology

The three-dimensional SDF structure and corresponding SMILES notation of MEHP were obtained from the PubChem database (Kim et al., 2023). Potential target genes of MEHP were predicted using the ChemBL (https://www.ebi.ac.uk/chembl/) (Mendez et al., 2019) and SwissTargetPrediction (http://www.swisstargetprediction.ch) (Daina et al., 2019) platforms, while NAFLD-related genes were identified through transcriptomic analysis. The overlap between MEHP targets and NAFLD-associated genes was then determined using the Lianchuan Bio cloud platform. These intersecting genes were subsequently uploaded to the STRING database (https://cn.string-db.org/) (Szklarczyk et al., 2023) to construct a protein–protein interaction (PPI) network. The resulting PPI network was further visualized in Cytoscape 3.10.1 (Shannon et al., 2003), and key candidate genes were screened using the CytoHubba plugin, leading to the identification of nine candidate genes. Finally, functional enrichment analyses, including KEGG and Gene Ontology (GO), were conducted on the overlapping gene set through the Microbioinformatics online platform (http://www.bioinformatics.com.cn/).

### 2.4 Identification of key features based on machine learning approaches

To identify key features, we applied multiple machine learning algorithms. First, Least Absolute Shrinkage and Selection Operator (LASSO) regression, a widely used data mining approach (Frost and Amos, 2017), was implemented using the R package “glmnet” to incorporate the features of the nine candidate genes into a diagnostic model. Specifically, the L1 regularization parameter was set to alpha = 1, and the optimal penalty parameter λ was determined using 10-fold cross-validation based on the minimum deviance criterion (lambda.min). Genes with nonzero regression coefficients were selected as candidate features. In addition, Support Vector Machine–Recursive Feature Elimination (SVM-RFE) (Nedaie and Najafi, 2018) was also applied for further feature refinement and selection. With the support of the R package “e1071”, the dataset was partitioned using 10-fold stratified cross-validation. In each fold, a linear-kernel SVM was iteratively trained, and features with the lowest contribution, as determined by feature weight coefficients, were recursively eliminated until the optimal feature subset was retained. Furthermore, a Random Forest (RF) model, a supervised ensemble learning method consisting of 500 decision trees (Diao et al., 2025), was constructed, and the MeanDecreaseAccuracy value of each gene was calculated to quantify the decrease in model prediction accuracy after permutation of that gene’s expression values. Genes with a MeanDecreaseAccuracy value greater than 0 were retained as positively contributing features, and the top six ranked genes were selected for visualization. Finally, the overlapping features identified by LASSO, SVM-RFE, and RF were defined as the candidate MEHP-associated NAFLD-related genes.

### 2.5 SHAP analysis

In this study, eleven machine learning methods were implemented to screen candidate MEHP-associated NAFLD-related genes, which markedly enhanced the accuracy and generalization capability of the predictive model. The applied algorithms comprised Logistic Regression (LR), Random Forest (RF), Gradient Boosting Machine (GBM), K-Nearest Neighbors (KNN), discriminant analysis, LASSO, Adaptive Boosting (AdaBoost.M1), Support Vector Machine (SVM), Partial Least Squares (PLS), Bayesian models, and neural networks.

Moreover, Shapley Additive Explanations (SHAP), an interpretable approach derived from cooperative game theory, was employed to evaluate the optimal model. This technique not only clarifies the underlying prediction mechanism but also quantitatively measures the contribution and relative importance of each feature. By incorporating SHAP analysis, a more transparent and in-depth understanding of the model’s decision-making process was achieved.

### 2.6 Development and validation of a binary logistic regression model

A binary logistic regression model was constructed based on the batch-corrected, integrated hepatic transcriptomic datasets GSE126848 and GSE24807 to estimate the regression coefficients of candidate MEHP-associated NAFLD-related genes and to generate individualized transcriptomic risk scores for each sample (Deng et al., 2024). To facilitate visualization and interpretation, a nomogram incorporating these key molecular features was developed using the “rms” R package. The performance of this exploratory risk model was evaluated through calibration curves, decision curve analysis (DCA), and receiver operating characteristic (ROC) curves. It should be noted that this model was established using hepatic transcriptomic data and should be regarded as an exploratory molecular classification tool, rather than a practical diagnostic instrument for routine clinical assessment of NAFLD.

### 2.7 Evaluation of immune cell infiltration in NAFLD

To further characterize the immune microenvironment, the CIBERSORT algorithm was applied to analyze gene expression data from NAFLD samples and to infer the relative proportions of infiltrating immune cell types (Newman et al., 2015). Subsequently, the associations between the expression of candidate MEHP-associated NAFLD-related genes and immune cell infiltration were examined using Spearman’s rank correlation analysis implemented in R. The correlation results were then visualized through the Lianchuan Bio Cloud Platform (Lyu et al., 2023), enabling a clearer interpretation of the interactions between key genes and immune cell composition.

### 2.8 Molecular docking

Molecular docking is a method used to predict the binding affinity and interaction mode between small molecules and target proteins. To further evaluate the potential interactions between MEHP and core target proteins, molecular docking analyses were conducted using Discovery Studio. The three-dimensional structures of BCL2L11, BMP2, ERBB2, and NFKB2 were retrieved from the Protein Data Bank (PDB) (Supplementary Table S1). Prior to docking, protein receptor structures were preprocessed by removing water molecules, deleting original ligands, adding hydrogen atoms, and performing necessary structural optimizations. The three-dimensional structure of MEHP (PubChem CID: 20393) was downloaded from the PubChem database and energy-minimized before docking. Docking analyses were then performed using the CDOCKER module in Discovery Studio. Active docking regions were defined based on the original ligand-binding sites or predicted binding pockets of each target protein. Docking conformations were ranked according to CDOCKER interaction energies, with lower values indicating more stable predicted interactions between the ligand and receptor. The conformation with the lowest CDOCKER interaction energy was selected for subsequent analyses, and hydrogen bonds, hydrophobic interactions, and relevant amino acid residues between MEHP and the target proteins were visualized using Discovery Studio Visualizer.

### 2.9 Cell culture

The AML12 mouse hepatocyte cell line and HepG2 cells were provided by the Chinese Academy of Sciences. AML12 cells were maintained in DMEM/F12 complete medium supplemented with 10% fetal bovine serum (FBS), 40 ng/mL dexamethasone, 1% ITS Liquid Media Supplement, and 1% penicillin–streptomycin solution. HepG2 cells were cultured in DMEM supplemented with 10% FBS and 1% penicillin–streptomycin solution. All cells were incubated at 37 °C in a humidified atmosphere containing 5% CO_2_.

### 2.10 Cell viability assay

To assess cell viability, a total of 8 × 10^3^ AML12 and HepG2 cells (suspended in 100 μL of complete DMEM/F12 and DMEM medium, respectively) were seeded into each well of a 96-well plate. After allowing the cells to adhere, AML12 and HepG2 cells were treated with MEHP across a concentration gradient of 0–800 μM (0, 1, 12.5, 25, 50, 100, 200, 400 and 800 μM). Based on previous studies indicating that 24-h treatment induces a pronounced cellular response (Chen et al., 2012; Bai et al., 2019), the cells were cultured for 24 h. Subsequently, 10 μL of CCK-8 reagent (Shangwei Bio, China) was added to each well, with blank control wells containing only medium and CCK-8 reagent to account for background absorbance. The plate was incubated in the dark at 37 °C with 5% CO_2_ in a humidified environment for 2 h, after which absorbance at 450 nm was measured using a microplate reader.

### 2.11 Biochemical indicator testing

Triglycerides (TG) and total cholesterol (TC) in the cells were measured according to the kit instructions. The kits were purchased from Nanjing Jiancheng Biotechnology Research Institute.

### 2.12 Green fluorescent lipid droplet assay

Lipid droplets were stained using a green fluorescent detection kit (Beyotime Inc., Shanghai, China) according to the manufacturer’s instructions. Cells were washed twice with PBS after removing the culture medium and then fixed with 4% paraformaldehyde for 15 min. Staining Solution (BDPY: Hoechst: Assay Buffer = 1:1:998) was applied, and cells were incubated at room temperature for 20 min in the dark. Green fluorescence was observed under a fluorescence microscope using 488 nm excitation.

### 2.13 Quantitative real-time PCR experiments

Both cell types were seeded into 6-well plates at a density of 5 × 10^6^ cells/mL and cultured for 24 h. The cells were then treated with MEHP at concentrations of 0, 1, 12.5, 25, and 50 μM for 24 h, with each experimental condition conducted in triplicate. At designated time points, cells were collected, and total RNA was isolated using the SteadyPure RNA Extraction Kit (Accurate Biology). The concentration and purity of the extracted RNA were assessed with a UV–visible spectrophotometer. Reverse transcription was then carried out using the Evo M-MLV Reverse Transcription Master Mix Kit (Ver. 2), which includes a genomic DNA removal step. The resulting cDNA was combined with 2× SYBR Green PCR Master Mix, and gene expression levels were quantified using a Bio-Rad CFX96 real-time PCR system. Fluorescence signals were recorded over 40 amplification cycles, with an annealing temperature set at approximately 60 °C. Primer sequences for the target genes are provided in Supplementary Table S2. Relative mRNA expression levels were calculated using the 2^^−ΔΔCt^ method, with GAPDH serving as the internal reference gene.

### 2.14 Western blot analysis

Cells were lysed in RIPA buffer and kept on ice for 30 min to ensure complete protein extraction. The lysates were then centrifuged at 12,000 × g for 15 min at 4 °C, after which the supernatants were carefully collected. Protein concentrations were measured using a BCA assay kit (Thermo Fisher Scientific, Waltham, MA, United States of America). Equal amounts of protein were combined with loading buffer and RIPA buffer, followed by heat denaturation at 95 °C for 15 min. Subsequently, 20 μg of protein per sample was separated by 10% SDS-PAGE and transferred onto PVDF membranes (Millipore, Billerica, MA, United States of America). The membranes were blocked with 5% skim milk for 2 h and then incubated overnight at 4 °C with primary antibodies. After washing with TBST, the membranes were incubated with HRP-conjugated goat anti-rabbit IgG (H&L) secondary antibody (1:5000) at room temperature for 1 h. Following three additional TBST washes, protein bands were visualized using an enhanced chemiluminescence detection system (Beyotime Inc., Shanghai, China). Images were acquired with a Bio-Rad imaging system, and band intensities were quantified using Image Lab software. GAPDH (36 kDa) served as the internal reference protein. The detailed information for the antibodies is provided in Supplementary Table S3.

### 2.15 Statistical analysis

All statistical analyses were conducted using GraphPad Prism version 10.1 (GraphPad, San Diego, CA, United States of America). Each *in vitro* experiment was performed with three independent biological replicates (n = 3). Differences among multiple groups were evaluated using one-way analysis of variance (ANOVA), followed by Tukey’s multiple-comparison test. A p-value of less than 0.05 was regarded as indicating statistical significance throughout the study.

## 3 Results

### 3.1 Identification of candidate genes

**FIGURE 1:**
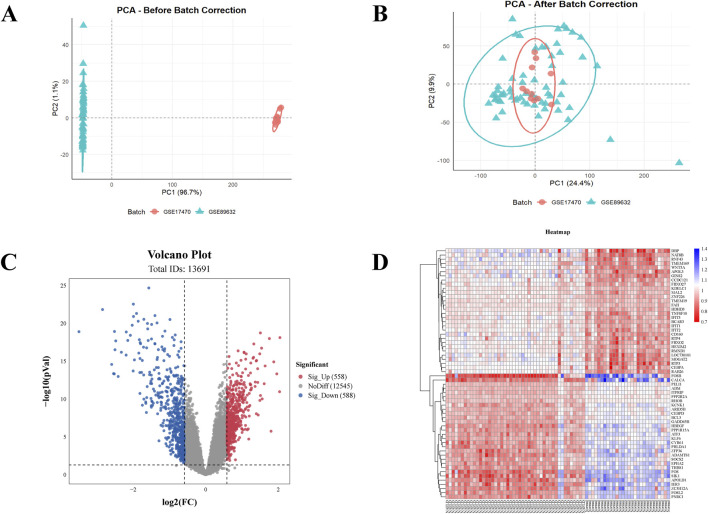
Identification of the DEGs in NAFLD. PCA of two datasets **(A)** before and **(B)** after batch effect correction. **(C)** A volcano map showing the DEGs in NAFLD patients compared to healthy controls. **(D)** heatmap of the top 30 upregulated and downregulated DEGs.

MEHP was first queried in the PubChem database to obtain its standardized structural data and detailed molecular properties. Potential targets of MEHP were then predicted using the ChEMBL and SwissTargetPrediction databases, resulting in a total of 1,131 candidate targets. To further identify genes associated with NAFLD, two transcriptomic datasets (GSE89632 and GSE17470) were combined and analyzed. After removing batch effects, principal component analysis (PCA) was conducted, and the distribution of samples is shown in Figures 1A,B. Differential expression analysis subsequently revealed 1,146 significantly altered genes, as illustrated in Figures 1C,D.

### 3.2 Network analysis of potential target interactions

**FIGURE 2:**
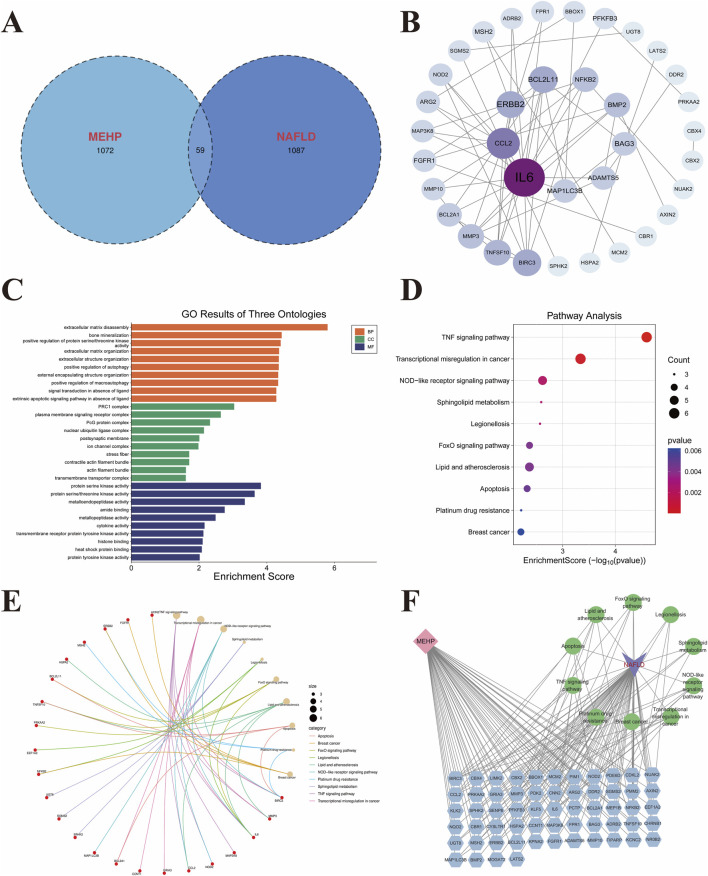
Identification of the key candidate targets and their biological roles in NAFLD. **(A)** Venn diagram exhibiting the overlap between genes associated with MEHP and those related to NAFLD; the shared genes were identified as the primary targets of MEHP in the context of NAFLD; **(B)** Protein–protein interaction (PPI) network of candidate genes constructed using STRING. **(C)** Bar chart illustrates the outcomes of Gene Ontology (GO) enrichment analysis for the identified candidate targets; **(D)** Bubble plot depicting the Kyoto Encyclopedia of Genes and Genomes (KEGG) pathway enrichment analysis of these candidate targets; **(E)** KEGG pathway–gene network of candidate targets. **(F)** Network linking MEHP, candidate targets, NAFLD, and enriched pathways.

By integrating DEGs with the predicted targets of MEHP, a total of 59 candidate targets were identified. These overlapping genes were regarded as potential targets involved in MEHP-associated NAFLD toxicity (Figure 2A). To further investigate the underlying interactions, a protein–protein interaction (PPI) network was established based on the STRING database. The constructed network was then imported into Cytoscape for visualization and analysis, where node connectivity and interaction strength were calculated and used to rank the importance of each node (Figure 2B).

### 3.3 Enrichment analysis of candidate genes

KEGG analysis indicated that MEHP may induce NAFLD through multiple key biological pathways, including the TNF signaling pathway, transcriptional misregulation in cancer, the NOD-like receptor signaling pathway, and sphingolipid metabolism, among others. Furthermore, based on the results of three major GO analyses, this study systematically elucidated the key functional interactions of the candidate MEHP-associated NAFLD-related genes. The significant enrichment of extracellular matrix organization and degradation, as well as autophagy, suggests that hepatic fibrosis and remodeling, together with programmed cell death, play central roles in disease progression (Figures 2C–E). Finally, Cytoscape software was used to construct a “toxin-target-pathway” network diagram for MEHP. As shown in Figure 2F, this diagram visually illustrates the potential connections among toxins, targets, and pathways.

### 3.4 Identification of candidate MEHP-associated NAFLD-related genes through machine learning

**FIGURE 3:**
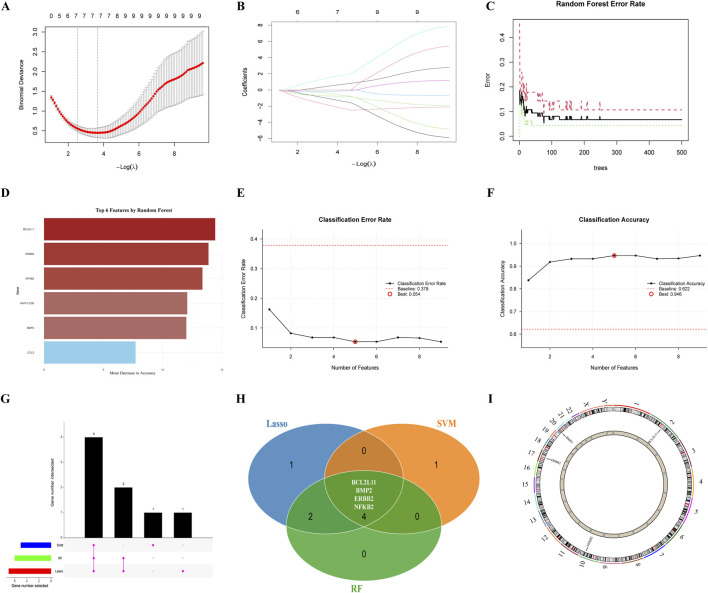
Identification of the candidate targets associated with MEHP-induced NAFLD by using machine-learning approaches. **(A,B)** Feature selection was performed using LASSO logistic regression method. The X-axis represents the number of genes retained at various λ (regu-larization) values, with nine genes selected at the optimal λ. **(C,D)** Random forest: relationship between tree number and error rate, ranking of candidate MEHP-associated NAFLD-related genes (importance >6). **(E,F)** SVM - RFE algorithm’s maximum accuracy and minimum error plots. **(G,H)** UpSet plot and Venn diagram showing overlapping genes among LASSO, SVM-RFE and Random Forest. **(I)** Chromosome localization of candidate MEHP-associated NAFLD-related genes.

To identify candidate MEHP-associated NAFLD-related genes, three machine learning approaches were applied. LASSO regression analysis selected seven candidate features from the training dataset (Figures 3A,B). The Random Forest model identified six important genes (Figures 3C,D), while the SVM-RFE method yielded five feature genes (Figures 3E,F).

By integrating the outputs of these three algorithms, four overlapping candidate MEHP-associated NAFLD-related genes were ultimately determined: BCL2L11, BMP2, ERBB2, and NFKB2 (Figures 3G,H). In addition, chromosomal mapping indicated that BCL2L11 is located on chromosome 2, NFKB2 on chromosome 10, ERBB2 on chromosome 17, and BMP2 on chromosome 20 (Figure 3I).

### 3.5 SHAP explanatory analysis

**FIGURE 4:**
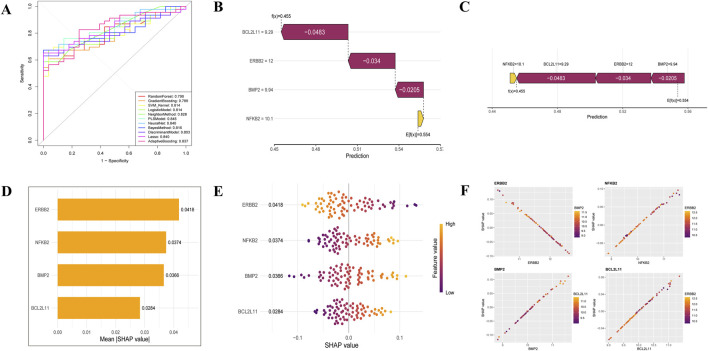
SHAP Explanatory Analysis. **(A)** Eleven machine learning algorithms used to identify candidate MEHP-associated NAFLD-related genes. **(B)** Waterfall plot and **(C)** force plot showing classification of individual samples based on integrated predictions from candidate MEHP-associated NAFLD-related genes. **(D)** Barplots showing the contribution magnitudes of the four genes in the PLS. **(E)** Beeswarm plot showing gene contribution distributions. **(F)** Scatter plots showing correlations between candidate MEHP-associated NAFLD-related genes and SHAP values.

To further assess the reliability of the selected candidate MEHP-associated NAFLD-related genes, eleven machine learning models—including LR, RF, GBM, KNN, discriminant analysis, LASSO, AdaBoost.M1, SVM, PLS, Bayesian approaches, and neural networks—were implemented (Figure 4A). Among these models, PLS achieved the highest predictive performance, as reflected by its superior AUC value. To explore the internal decision-making mechanism of the PLS model, SHAP values were computed using the “shapviz” R package, and features were ranked based on their contributions. Waterfall and force plots (Figures 4B,C) illustrated how the cumulative effects of the four genes influenced prediction outcomes at the individual sample level, providing a clearer interpretation of the model’s classification process. According to the bar plot results (Figure 4D), BMP2, ERBB2, and NFKB2 contributed most significantly to the predictive performance of the PLS model. Meanwhile, the heatmap (Figure 4E) underscored the pivotal role of ERBB2 in distinguishing between sample groups and indicated that all four candidate MEHP-associated NAFLD-related genes (BCL2L11, BMP2, ERBB2, and NFKB2) positively contributed to prediction outcomes. These observations suggest that alterations in the expression levels of these genes, relative to normal controls, are closely linked to disease occurrence. Furthermore, scatter plot analysis (Figure 4F) revealed positive correlations between SHAP values and the expression levels of BMP2, BCL2L11, and NFKB2, implying potential cooperative effects among these genes. In contrast, ERBB2 showed a negative association with SHAP values, suggesting a possible suppressive role in the model, which is consistent with previous findings.

### 3.6 Construction and evaluation of the logistic regression model

**FIGURE 5:**
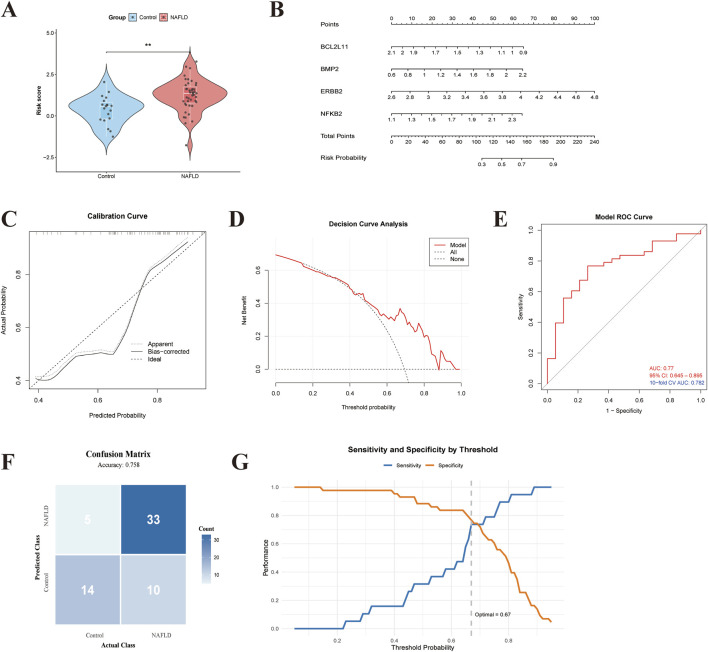
Candidate MEHP-associated NAFLD-related genes risk prediction model. **(A)** The risk scores calculated by the model for patients with NAFLD and healthy controls. **(B)** Logistic model for the prediction of NAFLD risk. **(C)** Calibration curves, **(D)** DCA, and **(E)** ROC evaluating the prediction efficacy of the nomogram. **(F)** Confusion matrix. **(G)** Sensitivity and specificity curves.

A binary logistic regression model was constructed using the four candidate MEHP-associated NAFLD-related genes. The final risk score formula was as follows: Risk score = (−1.9490 × BCL2L11) + (1.4544 × BMP2) + (1.6366 × ERBB2) + (1.7819 × NFKB2). Compared to the healthy control group, NAFLD samples exhibited higher transcriptomic risk scores (Figure 5A). Based on the expression levels of these four candidate genes, a nomogram was further developed to visualize this exploratory transcriptomic classification model (Figure 5B). The classification performance of the model was subsequently evaluated using calibration curves (Figure 5C), decision curve analysis (DCA) (Figure 5D), and receiver operating characteristic (ROC) curves. Ten-fold cross-validation showed an area under the curve (AUC) of 0.87 (Figure 5E). Additionally, the confusion matrix (Figure 5F), sensitivity curve, and specificity curve (Figure 5G) further indicated that this exploratory model, based on hepatic transcriptomic data, possesses a certain capacity to distinguish NAFLD samples from healthy controls. It should be noted that this model is not intended as a practical diagnostic tool for routine clinical NAFLD diagnosis, but rather as an exploratory molecular classification model derived from transcriptomic data. Detailed performance metrics of the model are presented in Supplementary Table S4.

### 3.7 Immune microenvironment of NAFLD patients

**FIGURE 6:**
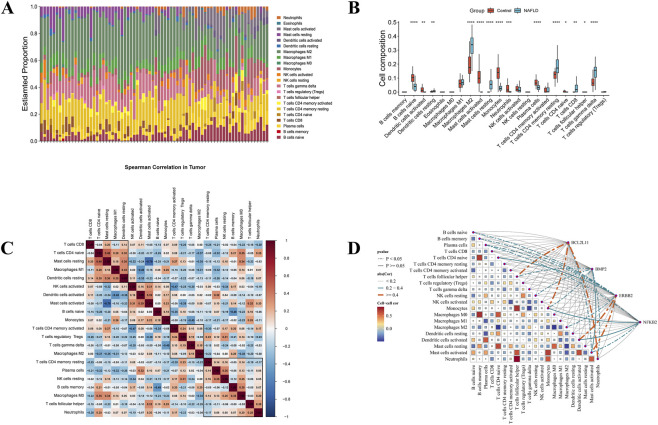
Analysis of immune cell infiltration in patients with NAFLD and healthy controls based on the GSE89632 and GSE17470 datasets. **(A,B)** Single-sample gene set enrichment analysis (CIBERSORT) was performed to quantify the infiltration levels of 22 immune cell populations in liver samples from patients with NAFLD and healthy controls using the GSE89632 and GSE17470 datasets. **(C)** Analysis of the interrelationships among different infiltrating immune cell types in NAFLD liver tissues. **(D)** Analysis of associations between candidate MEHP-associated NAFLD-related genes expression and immune cell infiltration levels in NAFLD liver tissues.

To explore alterations in the immune microenvironment associated with NAFLD, we first examined the distribution of 22 immune cell types between patients and healthy individuals (Figure 6A). Subsequently, a comparative analysis was conducted to quantify differences in immune cell abundance across the two groups. The results indicated that 14 immune cell subsets exhibited significant changes in NAFLD samples (Figure 6B). Specifically, the levels of naive B cells, M2 macrophages, activated and resting mast cells, monocytes, neutrophils, plasma cells, resting CD4 memory T cells, and gamma delta T cells were markedly elevated in NAFLD patients compared with controls. Furthermore, a Spearman correlation heatmap was generated to assess interactions among the 22 immune cell types (Figure 6C). Notably, activated CD4 memory T cells showed a strong negative correlation with M2 macrophages (cor = −0.70), implying that M2 macrophages may inhibit effector T cell activity through immunosuppressive mechanisms. In addition, correlation analysis between candidate MEHP-associated NAFLD-related genes and immune cell subsets (Figure 6D) revealed significant associations with multiple immune populations. Among these relationships, ERBB2 exhibited the strongest positive correlation with activated dendritic cells (cor = 0.60, p < 0.05). Collectively, these findings provide valuable insights into the interplay between MEHP-related factors and immune regulation in NAFLD.

### 3.8 Molecular docking of candidate MEHP-associated NAFLD-related genes

**FIGURE 7:**
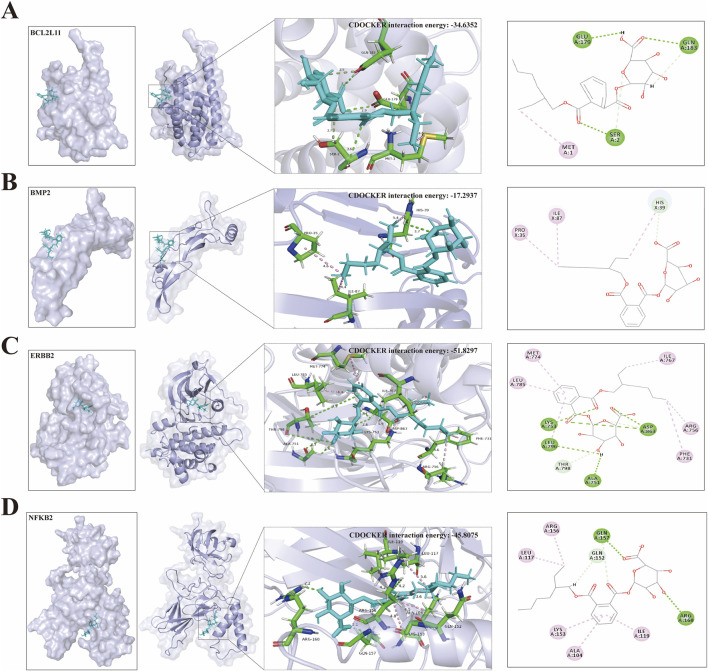
Molecular docking outcomes showing the binding affinity of MEHP with each target protein. **(A)** MEHP and BCL2L11. **(B)** MEHP and BMP2. **(C)** MEHP and ERBB2. **(D)** MEHP and NFKB2.

To further assess the potential binding interactions between MEHP and the four core target proteins—BCL2L11, BMP2, ERBB2, and NFKB2—molecular docking analyses were performed using the CDOCKER module in Discovery Studio. The results indicated that MEHP exhibited measurable docking interactions with all four target proteins (Figures 7A–D). Notably, ERBB2 displayed the lowest CDOCKER interaction energy at −51.8297, suggesting a relatively more stable predicted binding mode with MEHP. Further interaction analysis revealed that MEHP may form hydrogen bonds with amino acid residues THR-789, ALA-751, and ASP-863 in ERBB2, which likely contributes to the stability of the binding conformation.

### 3.9 Experimental validation of network toxicology

**FIGURE 8:**
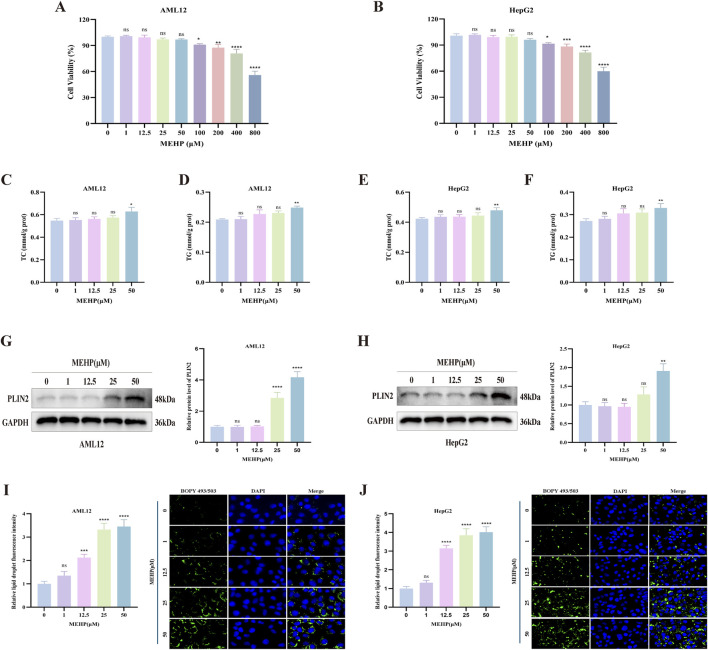
Experimental verification. **(A,B)** The CCK-8 assay was employed to assess the impact of varying intervention concentrations on cell viability (n = 3). **(C–F)** Levels of intracellular TG, TC (n = 3). **(G,H)** Protein expression level of PLIN2 (n = 3). **(I,J)** Representative fluorescence images show control cells and cells treated with MEHP at 1, 12.5, 25, and 50 μM, green fluorescence indicates lipid droplets. Scale bars = 12.5 μm *P < 0.05, **P < 0.01, ***P < 0.001, ****P < 0.0001.

Integrating results from network toxicology, machine learning, transcriptomic analyses, and molecular docking, BCL2L11, BMP2, ERBB2, and NFKB2 were identified as candidate targets potentially involved in MEHP-induced nonalcoholic fatty liver disease (NAFLD). To further validate these computational findings, *in vitro* experiments were conducted using AML12 and HepG2 cells. Cell viability was first assessed using the CCK-8 assay (Figures 8A,B). Results indicated that low concentrations of MEHP (0–50 μM) for 24 h had no significant effect on cell viability, whereas higher concentrations (100–800 μM) significantly reduced cell survival. Based on these findings, non-cytotoxic MEHP concentrations (0, 1, 12.5, 25, and 50 μM) were selected for subsequent experiments.

Lipid accumulation in cells treated with MEHP for 24 h was then evaluated. Biochemical assays revealed that within the 0–25 μM range, intracellular TG and TC levels were not significantly different from controls. However, at 50 μM MEHP, both TG and TC levels were markedly elevated in both cell types (Figures 8C–F). Intracellular lipid deposition was further assessed using green fluorescent lipid droplet staining. In control cells, no appreciable lipid accumulation was observed. Compared to controls, 1 μM MEHP induced slight lipid increase without statistical significance, whereas 12.5, 25, and 50 μM MEHP treatment led to pronounced lipid deposition in both cell types, with significant differences relative to controls (P < 0.05) (Figures 8I,J). To further confirm MEHP-induced lipid accumulation, the expression of the lipid droplet-associated protein Plin2 was examined. Results showed that PLIN2 protein levels were significantly elevated in both cell types following 25 and 50 μM MEHP treatment, providing additional evidence that MEHP promotes intracellular lipid deposition (Figures 8G,H).

**FIGURE 9:**
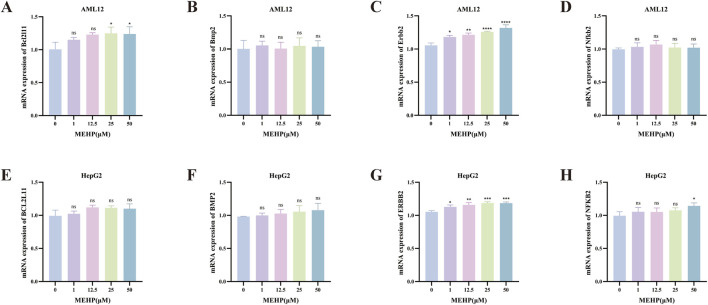
MEHP Modulates Gene Expression. **(A–H)** The mRNA expression levels of *Bcl2l11, Bmp2, Erbb2 and Nfkb2* were analyzed using quantitative real-time polymerase chain reaction (qRT-PCR) (n = 3). *P < 0.05, **P < 0.01, ***P < 0.001, ****P < 0.0001.

The transcriptional changes in candidate genes were further analyzed using real-time quantitative PCR. The results showed that *ERBB2* mRNA was significantly upregulated in human HepG2 cells, while *Erbb2* mRNA was significantly upregulated in mouse AML12 cells following MEHP exposure, suggesting a potential role of *ERBB2/Erbb2* in MEHP-induced hepatocellular injury. In AML12 cells, *Bcl2l11* mRNA was markedly increased after exposure to 25 and 50 μM MEHP, whereas no significant change in *BCL2L11* mRNA was observed in HepG2 cells. In contrast, *BMP2/Bmp2* and *NFKB2/Nfkb2* mRNA levels showed no significant alterations in either cell type (Figures 9A–H). Given the stable and consistent upregulation of *ERBB2/Erbb2* across both cellular models, this gene was selected as the primary target for subsequent experimental validation.

**FIGURE 10:**
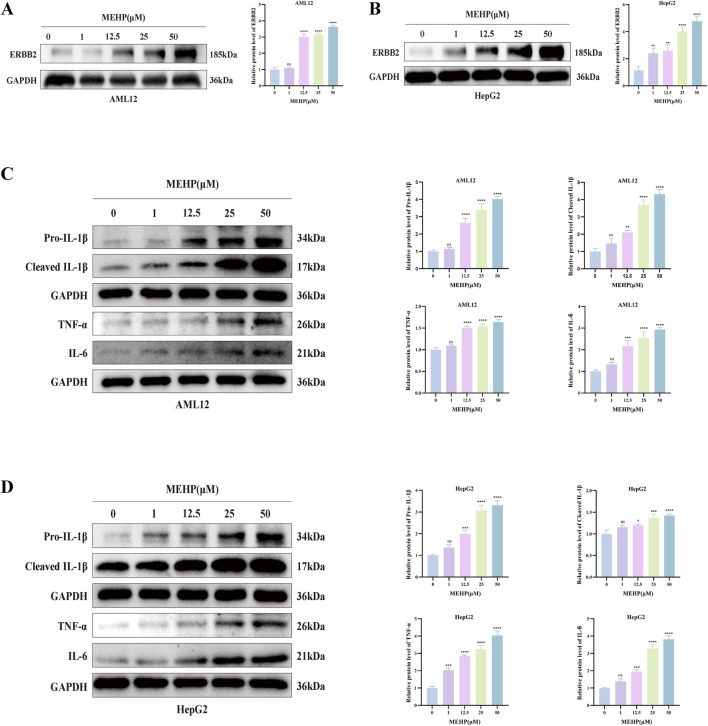
MEHP Modulates Protein Expression. **(A,B)** Protein expression level of ERBB2 (n = 3). **(C,D)** Protein expression level of IL-1β, TNF-α, IL-6 (n = 3). *P < 0.05, **P < 0.01, ***P < 0.001, ****P < 0.0001.

Consistent with the mRNA results, Western blot analyses further confirmed that MEHP treatment markedly increased ERBB2 protein expression (Figures 10A,B). Moreover, compared to the control group, MEHP exposure increased the intracellular protein expression levels of TNF-α, IL-6, and IL-1β, suggesting that MEHP treatment may be accompanied by inflammation-related molecular alterations, with the effects most pronounced at 12.5–50 μM concentrations (Figures 10C,D). These findings indicate that MEHP exposure induces inflammation-related molecular alterations in hepatocytes and suggest that ERBB2 upregulation may be closely associated with this process, providing an experimental basis for further investigation into ERBB2-mediated MEHP-induced inflammatory injury mechanisms.

## 4 Discussion

MEHP is a bioactive metabolite of DEHP (Albro et al., 1982) and a prototypical environmental endocrine-disrupting chemical (EDC) widely present in plastic products and consumer goods. Humans can be exposed to MEHP through food, air, and water (Yao et al., 2025; Li et al., 2025). Studies have shown that DEHP is rapidly hydrolyzed to MEHP in human organs and rat liver, and MEHP is subsequently metabolized in the liver by CYP enzymes to form oxidized and de-alkylated products (Choi et al., 2012). Furthermore, studies using MEHP-AF as a fluorescent tracer indicate that MEHP is rapidly absorbed via the rat intestine and primarily distributed in the liver, kidneys, and testes (Yuan et al., 2022). At the cellular level, MEHP exposure induces lipid accumulation in BRL-3A and HepG2 cells, elevates liver function markers (ALT/AST), and disrupts lipid metabolism, ultimately promoting NAFLD development (Zhang et al., 2019; Bai et al., 2019). Therefore, elucidating the molecular mechanisms underlying MEHP-induced NAFLD is of critical importance.

NAFLD is primarily a chronic liver disorder driven by metabolic dysregulation, characterized by abnormal lipid accumulation in hepatic tissue, particularly elevated TC and TG levels (Jin et al., 2018; Wang et al., 2021). Epidemiological studies have consistently demonstrated a close association between lipid abnormalities and NAFLD. Elevated TG levels are not only strongly correlated with hepatic lipid accumulation but are also significantly higher in NAFLD patients compared to healthy controls; delayed postprandial TG elevation is likewise associated with increased NAFLD risk, suggesting that TG is a key metabolic marker of disease progression (Wu et al., 2016; Hou et al., 2021). Plin2 also plays an important role in hepatocellular steatosis by contributing to intracellular lipid droplet formation, blocking the access of lipases to neutral lipids, thereby inhibiting triglyceride hydrolysis and maintaining lipid droplet stability (Listenberger et al., 2007). In this study, MEHP treatment significantly increased intracellular TG and TC levels, accompanied by a dose-dependent upregulation of Plin2, indicating that MEHP promotes hepatocellular lipid accumulation and contributes to NAFLD-like pathological changes.

Mechanistically, KEGG enrichment analysis of MEHP-related differentially expressed genes revealed significant enrichment in the TNF signaling pathway and other inflammation-related pathways, suggesting that inflammatory responses may represent a potential mechanism by which MEHP promotes NAFLD progression. Further integration of machine learning and molecular docking analyses identified ERBB2 as a potential core gene mediating MEHP-induced inflammatory responses. ERBB2 is a receptor tyrosine kinase belonging to the EGFR/ERBB/HER kinase family (Yarden and Sliwkowski, 2001). Previous studies indicate that ERBB2/HER2 signaling not only regulates cell proliferation and differentiation but can also activate the NF-κB pathway via the canonical IKKα/β-NF-κB axis, thereby modulating the expression of inflammation-related genes and cytokines; inhibition of ERBB2 reverses this process, suggesting that ERBB2 may have a functional association with inflammatory signaling (Jiang et al., 2014; Merkhofer et al., 2010). Moreover, ERBB family signaling is also implicated in inflammation and immune microenvironment remodeling (Rahman et al., 2019). Together, these findings suggest that ERBB2 may function not only as a regulator of cell growth but also as a mediator of amplified inflammatory signaling in response to MEHP exposure.

TNF-α is a prototypical pro-inflammatory cytokine with broad functions in tissue homeostasis and inflammatory responses. It can activate the NF-κB signaling pathway through binding to TNF receptors, promoting transcription of inflammatory genes and immune responses, while NF-κB activation in turn enhances TNF-α expression, forming a positive feedback loop (Aggarwal et al., 2012; Beutler et al., 2007). Studies have demonstrated that TNF-α is a key driver of NAFLD pathogenesis and its progression to hepatocellular carcinoma (Vachliotis et al., 2023; Cobbina and Akhlaghi, 2017). Elevated serum TNF-α levels serve as an inflammatory biomarker for NAFLD and can help distinguish early-stage lesions from healthy controls (Mounika et al., 2025; Afrisham et al., 2025). Meta-analyses further indicate that increased TNF-α levels are significantly associated with NAFLD, NASH, and liver fibrosis, highlighting its critical role in disease initiation and progression (Duan et al., 2022). Systematic reviews suggest that TNF-α contributes to NAFLD pathophysiology by promoting hepatic inflammation, insulin resistance, and cellular stress (Vachliotis and Polyzos, 2023). During NAFLD progression, TNF-α can also activate the NLRC4 inflammasome and promote its mitochondrial translocation, inducing IL-18 and IL-1β release and hepatocyte pyroptosis, thereby exacerbating hepatic inflammation and disease progression (Chen and Ma, 2019).

Immune infiltration analyses further suggest that ERBB2 may be associated with dendritic cell (DC) activation. Hepatic DCs are a key component of innate immunity (Chen et al., 2021), and their sustained activation in chronic liver disease can drive destructive inflammatory responses, aggravating tissue damage and fibrosis (Ma et al., 2023). Previous studies have shown that the ML2 domain of ERBB2 can induce DC maturation and enhance their functional capacity (Zhong et al., 2005; Xu et al., 2007; Münz et al., 2005). Thus, combined with our immune infiltration results, ERBB2 may be involved in the production of inflammation-related proteins such as TNF-α and hepatic immune microenvironment remodeling by modulating DC activation.

In addition to TNF-α, IL-6 and IL-1β are also important pro-inflammatory cytokines in NAFLD progression. Multiple inflammatory mediators, including IL-6, IL-1β, and TNF-α, have been implicated in NAFLD development and progression (Rafaqat et al., 2024). IL-6 is a pleiotropic cytokine with complex roles in inflammatory responses (Schmidt-Arras and Rose-John, 2016). Xiao et al. reported that activation of the IL-6/STAT3 signaling axis promotes NAFLD progression and exacerbates hepatic inflammation (Park et al., 2023). Moreover, inhibition of ERBB2 significantly attenuates IL-6–mediated MAPK activation, suggesting that ERBB2 may amplify IL-6–associated inflammatory signaling (Qiu et al., 1998). IL-1β is likewise a critical pro-inflammatory cytokine in NAFLD progression, with elevated levels significantly associated with NAFLD, NASH, and liver fibrosis (Duan et al., 2022). NLRP3 inflammasome/caspase-1–mediated IL-1β activation further promotes hepatic inflammation and disease progression (Mirea et al., 2018). Animal studies also demonstrate that IL-1β deficiency markedly alleviates diet-induced hepatic inflammation and steatosis, indicating that IL-1β is not only an inflammatory biomarker but may also play a pathogenic role in NAFLD development (Tilg and Moschen, 2011).

Based on the above bioinformatics analyses and literature evidence, this study further validated the findings through *in vitro* experiments. In these experiments, cells were exposed to MEHP at 0, 1, 12.5, 25, and 50 μM for 24 h. Biomonitoring studies indicate that DEHP metabolites are ubiquitous in human urine, and as the primary hydrolytic metabolite of DEHP, MEHP is present at concentrations far below those used *in vitro*. For instance, analysis of 53 adult samples from northern Bavaria, Germany, revealed a median urinary MEHP concentration of approximately 10.3 μg/L (range <0.5–177 μg/L), corresponding to roughly 0.03–0.04 μM (Koch et al., 2003). Although concentrations vary across countries and regions, the majority of human populations exhibit urinary DEHP metabolite levels in the tens of μg/L range (Wang et al., 2019; Kato et al., 2004). In a previous *in vitro* study, Yang et al. treated human hepatocellular carcinoma HepG2 cells with 6.25–100 μM MEHP and found that MEHP at concentrations ≥25 μM induced oxidative DNA damage after 24 h of exposure and was associated with alterations in the p53-mediated mitochondria-dependent apoptotic pathway (Yang et al., 2012). Therefore, the 1–50 μM dose range used in the present study is, from an experimental design perspective, partially comparable to the micromolar concentrations used in previous *in vitro* mechanistic studies of MEHP. However, these concentrations are substantially higher than typical urinary MEHP levels reported in biomonitoring studies of the general population. Thus, the present findings should be interpreted primarily as experimental evidence of MEHP-related molecular mechanisms under short-term *in vitro* conditions, rather than being directly extrapolated to biological effects under real-world human environmental exposure scenarios. Future studies using lower concentrations, long-term exposure models, and experimental systems that more closely reflect human exposure conditions are needed for further validation. In addition, Western blot analyses further validated the *in vitro* dose-response experiments. Results showed a marked increase in ERBB2 protein expression following MEHP exposure, suggesting that ERBB2 may participate in MEHP-induced hepatocellular injury and inflammatory responses. Concurrently, cellular assays showed increased levels of inflammation-related proteins, including TNF-α, IL-6, and IL-1β, suggesting that MEHP exposure may be accompanied by inflammation-related molecular alterations. Although direct evidence that ERBB2 drives TNF-α expression is currently lacking, previous studies suggest potential interactions or cross-regulation between ERBB2 signaling and TNF-α–related inflammatory pathways (Ceran et al., 2012). Therefore, given the significant enrichment of the TNF signaling pathway in the KEGG analysis, the identification of ERBB2 as a candidate hub gene through machine-learning and molecular docking analyses, and the observed increase in ERBB2 protein expression following MEHP exposure by Western blotting, we propose that ERBB2 may participate in MEHP-associated alterations in inflammatory signaling. However, the proposed MEHP–ERBB2–inflammation axis remains a mechanistic hypothesis supported by bioinformatic analyses, molecular docking, and preliminary *in vitro* validation.

Nevertheless, this study has several limitations. First, although MEHP exposure was observed to increase ERBB2 protein expression and was accompanied by elevated levels of inflammation-related proteins, including TNF-α, IL-6, and IL-1β, these proteins were primarily detected by Western blotting in cell lysates. Therefore, the results reflect changes in intracellular or total protein expression rather than secreted cytokine levels in the culture supernatant. Moreover, the causal relationship between ERBB2 upregulation and these inflammation-related molecular alterations has not been definitively established through ERBB2 knockdown, overexpression, or specific inhibitor-based interventions. Second, direct validation of the interaction between MEHP and ERBB2 using CETSA, SPR, or other protein–ligand binding assays has not been performed, which is crucial for elucidating the molecular mechanism. Additionally, this study focused exclusively on MEHP and did not consider other DEHP metabolites, including MEHHP, MEOHP, MECPP, or total ΣDEHP, limiting the generalizability of the findings to human DEHP exposure and NAFLD risk. Furthermore, the machine learning approaches employed in the bioinformatics analyses may be prone to overfitting, potentially affecting the reliability and generalizability of the predictions. Notably, the MEHP concentrations used *in vitro* were higher than typical human exposure levels, which may affect the physiological relevance of the results and warrant further investigation in long-term, low-dose exposure models. Finally, the role of the MEHP–ERBB2–inflammation axis in NAFLD pathogenesis requires further validation in animal studies and larger clinical cohorts.

**FIGURE 11:**
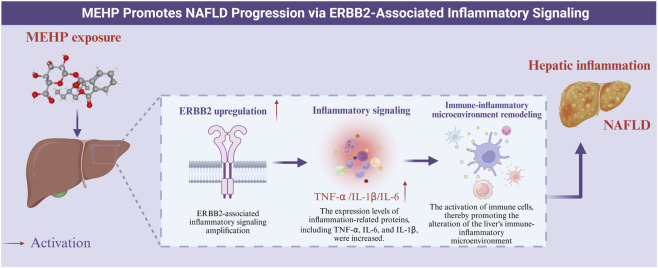
MEHP promotes NAFLD progression via ERBB2-Associated inflammatory signaling.

In summary, our findings suggest that MEHP exposure promotes hepatocellular lipid accumulation and inflammation-related molecular alterations, accompanied by increased ERBB2 expression. Integrating bioinformatic analyses, immune infiltration analysis, molecular docking, and preliminary *in vitro* validation, this study proposes that ERBB2 may be involved in MEHP-associated inflammatory alterations. ERBB2 upregulation after MEHP exposure, together with altered expression of inflammation-related proteins including TNF-α, IL-6, and IL-1β, may form a potential MEHP–ERBB2–inflammation-related molecular network that participates in remodeling the hepatic immune-inflammatory microenvironment and promoting NAFLD progression. The proposed mechanism is summarized in Figure 11. Nevertheless, this proposed MEHP–ERBB2–inflammation axis remains a mechanistic hypothesis derived from multi-omics analyses, molecular docking, and preliminary *in vitro* experiments, and requires further validation through functional studies.

## 5 Conclusion

This study employed an integrative approach combining network toxicology, machine learning, molecular docking, immune infiltration analysis, and *in vitro* experiments to preliminarily explore the potential molecular mechanisms underlying MEHP-associated NAFLD. The results identified ERBB2 as a candidate hub gene worthy of further investigation. MEHP exposure induced hepatocellular lipid accumulation and increased ERBB2 protein expression, accompanied by elevated levels of inflammation-related proteins, including TNF-α, IL-6, and IL-1β. These findings suggest that ERBB2 may be associated with MEHP exposure-related alterations in inflammation-related proteins and remodeling of the hepatic immune-inflammatory microenvironment, and may participate in NAFLD-related pathological processes. Therefore, the proposed MEHP–ERBB2–inflammation axis should be regarded as a preliminary mechanistic hypothesis. Future studies involving ERBB2 functional intervention experiments and direct binding validation assays are required to further confirm this proposed mechanism.

## Data Availability

The original contributions presented in the study are included in the article/Supplementary Material, further inquiries can be directed to the corresponding authors.
