# Supplementary material for: Hepatic lipid accumulation induced by mono-(2-ethylhexyl) phthalate and potential ERBB2-associated inflammatory signaling in NAFLD
Source: Front Pharmacol. 2026 Jun 25;17:1848121. doi: 10.3389/fphar.2026.1848121 (PMC13346091; doi:10.3389/fphar.2026.1848121)
Supplement: Supplementary file 1 [file Table1.docx]

**Table S1 Information of target proteins used for molecular docking**

| Protein | PDB ID | CDOCKER interaction energy |
| --- | --- | --- |
| BCL2L11 | 1PQ1 | -34.6352 |
| BMP2 | 6OML | -17.2937 |
| ERBB2 | 3PP0 | -51.8297 |
| NFKB2 | 7VUQ | -45.8075 |

**Table S2 qRT-PCR primer information**

| **Gene** | **Primer sequence (5′–3′)** |
| --- | --- |
| **Mouse primers** | |
| *Bcl2l11-F* | GCCAAGCAACCTTCTGATGT |
| *Bcl2l11-R* | GCGGTTCTGTCTGTAGGGAG |
| *Bmp2-F* | GCGTCAAGCCAAACACAAAC |
| *Bmp2-R* | GAGTTCAGGTGGTCAGCAAG |
| *Erbb2-F* | ACATGCTTCGCCACCTCTAC |
| *Erbb2-R* | AGCTGAGTCCCTCTCACGAT |
| *Nfkb2-F* | GGCCTGAGAGGGATACCCAA |
| *Nfkb2-R* | TAGCAGCAGAGTCTTCACCTTG |
| *GAPDH-F* | TGTGTCCGTCGTGGATCTGA |
| *GAPDH-R* | TTGCTGTTGAAGTCGCAGGAG |
| **Human primers** | |
| *BCL2L11-F* | ACTCTCGGACTGAGAAACGC |
| *BCL2L11-R* | CCTGCCTCATGGAAGCTTGT |
| *BMP2-F* | GGAACGGACATTCGGTCCTT |
| *BMP2-R* | CACCATGGTCGACCTTTAGGA |
| *ERBB2-F* | AGCAGAGGATGGAACACAGC |
| *ERBB2-R* | CTGGTAACTGCCCTCACCTC |
| *NFKB2-F* | GATCCACGTCGACACCGTT |
| *NFKB2-R* | TAGGGGCCATCAGCTGTTTC |
| *GAPDH-F* | AATGGGCAGCCGTTAGGAAA |
| *GAPDH-R* | GCCCAATACACCACAAATCAGAG |

**Table S3 Primary antibody information**

| **Antibody** | **Host species** | **Supplier** | **Catalog number** | **Dilution** |
| --- | --- | --- | --- | --- |
| ERBB2 | Rabbit | Proteintech | 18299-1-AP | 1:2000 |
| PLIN2 | Rabbit | Proteintech | 230274F4 | 1:1000 |
| IL-1β | Rabbit | Proteintech | 16806-1-AP | 1:1000 |
| TNF-α | Rabbit | Proteintech | 17590-1-AP | 1:1000 |
| IL-6 | Rabbit | Proteintech | 21865-1-AP | 1:1000 |
| GAPDH | Rabbit | Proteintech | 10494-1-AP | 1:10000 |

**Table S4 Performance metrics of the logistic regression model**

| **Model** | **Performance metric** | **Value** |
| --- | --- | --- |
| Logistic regression model | AUC | 0.77 |
|  | 10-fold Cross-validated AUC | 0.782 |
|  | Accuracy | 0.758 |
|  | Sensitivity | 0.737 |
|  | Specificity | 0.767 |
|  | Optimal cutoff value | 0.67 |
